# Factors Associated With an Increased Risk of Facial Malformations

**DOI:** 10.7759/cureus.41641

**Published:** 2023-07-10

**Authors:** Saad Slah-Ud-Din, Kunza Ali, Syed Muhammad Mahd, Samaha Nisar, Omar Nisar

**Affiliations:** 1 Internal Medicine, Shalamar Medical & Dental College, Lahore, PAK

**Keywords:** facial malformations, cleft lip and palate, associated factors, consanguinity, birth defects

## Abstract

Background

Facial anomalies comprise a significant component of birth defects, with oral clefts being the second most common entity in this group. All organ systems within the body can be affected by congenital anomalies, mostly affecting the musculoskeletal system. Birth defects are among the leading causes of infant mortality and morbidity around the world.

Objectives

To find the factors associated with an increased risk of facial malformations so that steps for improving preventive measures can be taken.

Methodology

This was a cross-sectional study in which the data were collected from the files of infants admitted to the pediatric department. Data regarding the type of congenital anomaly, maternal investigations done during pregnancy, maternal history of medication, diabetes, hypertension, radiation exposure, smoking, and alcohol history, and family history of congenital anomalies was collected from the files of neonates and from the pediatrician. In the case of unanswered questions, the parent was contacted after 10 days with their consent.

Results

Of the sample size of 259 children (males: 132; females: 127), 68 (26%) had a cleft lip, 69 (27%) had a cleft palate, 110 (42%) had both cleft lip and palate, five (2%) had a cleft lip with nasal deformity, five (2%) had a cleft lip and palate with nasal deformity, and two (1%) had hypertelorism. Eight percent of neonates with craniofacial malformations had a family history of congenital malformations; 80.7% of neonates had a history of parental consanguinity; and 19.3% were unrelated. In regard to the mothers, 41.3% of the mothers had diabetes, 4% had hypertension, 4% had both gestational diabetes and hypertension, and 55% had neither of these diseases. Of the 55% of mothers with neither disease, 75% were married to their cousins, while 25% were not married within the family.

Practical implications

This study, highlighting the major factors contributing to the incidence of congenital facial malformations, will educate the community and establish awareness among the younger generation of the top causes of anomalies, therefore making a huge impact on increasing efforts to reduce the prevalence of congenital anomalies.

Conclusion

Defects of both the cleft lip and palate had the highest prevalence of facial malformations among study subjects (110 patients (42%)). Parental consanguinity is one of the leading factors associated with an increased risk of facial malformations.

## Introduction

Facial malformations are structural anomalies, prevalent in 2%-3% of the global population as a result of a multifactorial cause, that affect the body and are notable at birth and also recognizable while intrauterine [1]. Facial anomalies impact a long-standing, undesirable follow-up on articulation and appearance, which proves to be adversely affecting individuals, leading to a narrowed life span and increasing challenges as the child progresses to adulthood [2].

Facial defects make up a noteworthy portion of accumulative birth anomalies [3] and constitute the foremost cause of infant mortality and morbidity globally [4]. The frequency of anomalies differs from region to region, with the Asian population marking the most prevalent territory in the world and the African population being the least affected [5].

Congenital defects affecting a newborn are most strikingly apparent in the musculoskeletal system of the body but are not just comprised of bony alterations but also affect other systems, for which the cardiovascular and gastrointestinal systems are remarkable [6]. The rehabilitation of an affected child involves a multidisciplinary approach, including psychological counseling [7].

The increasing prevalence of facial anomalies is directly linked to a number of risk factors, including an immensely high level of parental consanguinity as well as multiple other factors, including inconsistent prenatal evaluation, medications, and the lifestyle of the mother during the gestational period [8,9].

The objective of our study was to find the factors associated with an increased risk of facial malformations so that steps for improving preventive measures could be taken.

## Materials and methods

A descriptive retrospective cross-sectional study was carried out after receiving ethical approval from Shalamar Medical & Dental College's Institutional Review Board, Lahore, Pakistan (SMDC-IRB/AL/17/2020).

The purposeful sampling technique was applied, and a sample size of 259 children was calculated. The data were collected from Shalamar Hospital, General Hospital, and Children's Hospital, all three hospitals situated in Lahore, Pakistan, as there was no subject participation. The data were collected in a one-year timeframe between January 2020 and January 2021 on a questionnaire designed with modifications to the validated questionnaire (Appendix A). Data were analyzed, and frequencies and percentages were calculated and tabulated.

The data were collected regarding the type of anomaly, maternal investigations during pregnancy, maternal medications, radiation exposure, smoking and alcohol history, and family history of congenital anomalies. Babies born preterm and babies born with craniofacial anomalies to mothers with any malignancy or hepatitis B or C were excluded from the study.

## Results

Of the sample size of 259 children, 68 (26%) had a cleft lip, 69 (27%) had a cleft palate, 110 (42%) had both cleft lip and palate, five (2%) had a cleft lip with nasal deformity, five (2%) had a cleft lip and palate with nasal deformity, and two (1%) had hypertelorism (Table 1).

**Table 1:** The percentage of malformations identified among the study subjects

	f	%
Cleft lip	68	26%
Cleft palate	69	27%
Cleft lip and palate	110	42%
Cleft lip and nasal deformity	5	2%
Cleft lip and palate and nasal deformity	5	2%
Hypertelorism	2	1%

**Figure 1:**
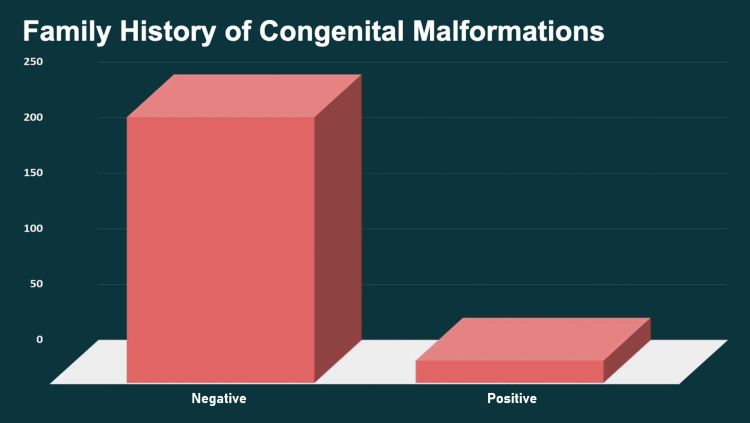
Family history of congenital malformations

Twenty-one (8%) of the neonates with craniofacial malformations had a family history of congenital malformations (Figure 1).

**Figure 2:**
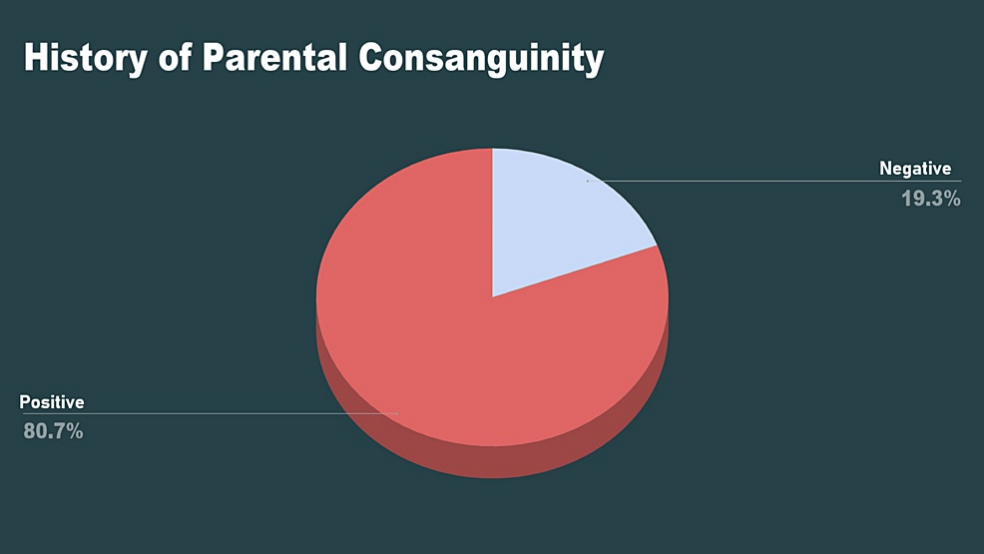
History of parental consanguinity

Two hundred and nine (80.7%) of the neonates had a history of parental consanguinity, whereas 50 (19.3%) were unrelated (Figure 2).

**Figure 3:**
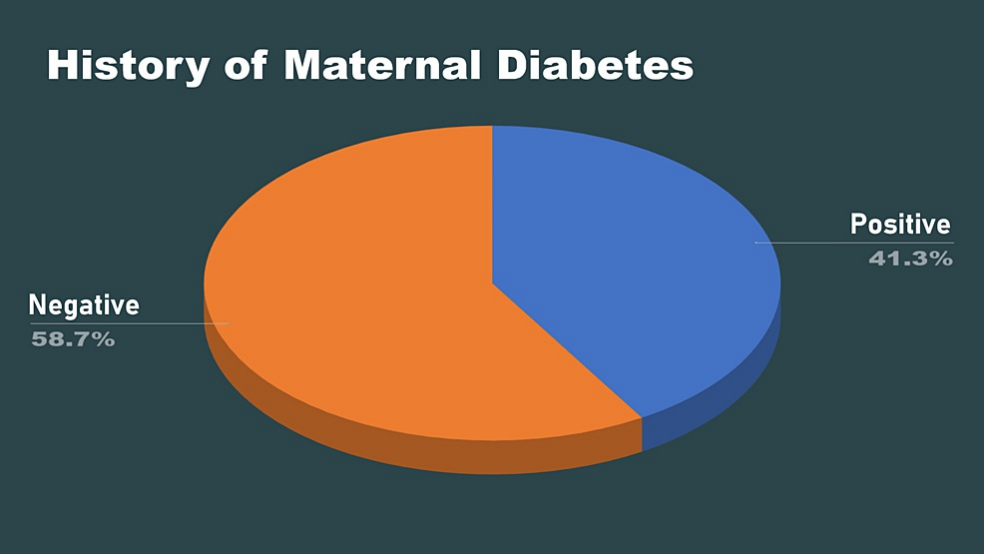
History of maternal diabetes

In regard to the mothers, 147 (57.1%) of the mothers had a history of radiation exposure; 107 (41.3%) of the mothers had diabetes (Figure 3).

Ten mothers (4%) had hypertension; 10 (4%) had both gestational diabetes and hypertension; and 142 (55%) had neither of these diseases. Of the 142 (55%) mothers with neither disease, 107 (75%) were married to their cousins, while 35 (25%) were not married within the family.

**Figure 4:**
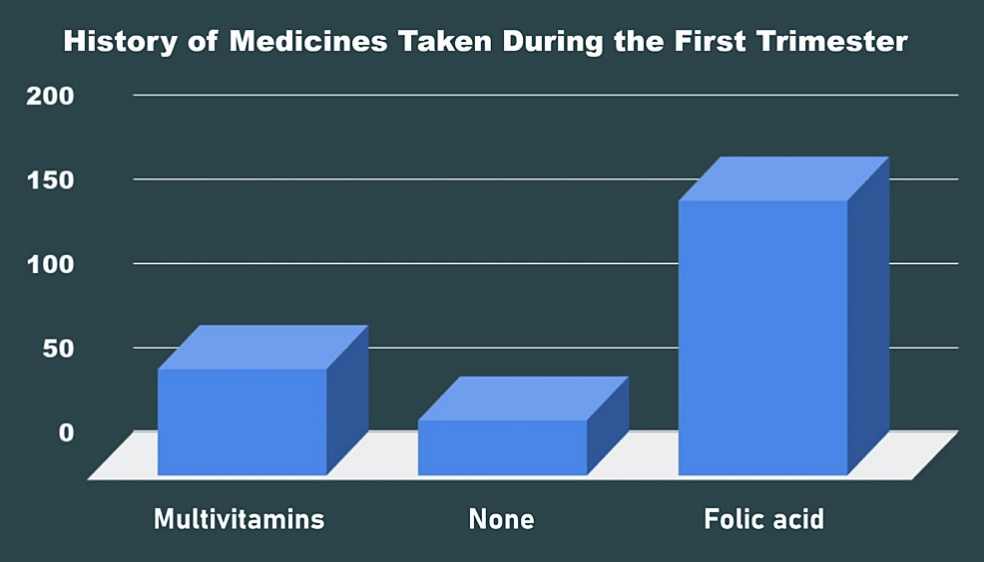
History of medicines taken by the mothers during the first trimester

One hundred and sixty-three (62.93%) of the gravid mothers took folic acid during the gestational period, 63 (24.32%) took multivitamins, and 33 (12.74%) took no medication during pregnancy (Figure 4).

Among all subjects, there was no history of maternal smoking, maternal alcohol consumption, or any history of maternal infections.

## Discussion

Facial malformations constitute a major portion of congenital defects, and orofacial clefts comprise more than one-third of the most common abnormalities [10]. There is much evidence worldwide of prevalent craniofacial anomalies, as Europe highlights a major 23.9 abnormalities per 1,000 births in a tertiary care setting [11]. Similar studies in a tertiary care hospital and the State University Teaching Hospital in Enugu, Nigeria, during the periods of 2007-2011 and 2015-2018, respectively, have been significant for the occurrence of congenital anomalies, marking prevalences of 2.8% and 1.7%, respectively. The most common system affected by the anomaly was found to be the musculoskeletal system (45.2%), followed by the central nervous system (34.9%) [12,13]. Immense progress in the discovery and study of craniofacial malformations has sparked interest in this field of scientific research [14].

Similarly, a prospective investigation held in Iran among 3,529 newborns over a course of 12 months revealed 109 neonates congenitally malformed, which in further scrutiny revealed a high 7% prevalence of malformation from consanguineous marriages as compared to a low 2% from non-consanguineous marriages [15].

A study executed at King Faisal Specialist Hospital and Research Center, Riyadh, Saudi Arabia, comprising 447 craniofacial malformed patients, was crucial in showing cleft palate association (56.8%), followed by cleft lip with cleft palate (32.9%) and cleft lip (20.5%), all of which were more pronounced in parents reserving consanguineous marriages [16]. Another study carried out at King Faisal Specialist Hospital and Research Center, Riyadh, Saudi Arabia, between 1999 and 2009 provided evidence of consanguinity between the parents of affected infants in 56.8% of the cases [17].

A research inquiry based on Palestinians was also significant, with a high 53% consanguinity rate leading to congenital malformations, as the recurrence of clefts in siblings was also notably high in consanguineous parents [18]. In research carried out in Pakistan on 3,210 hospital admissions, 7% were reported to be suffering from a congenital abnormality comprising musculoskeletal and central nervous system associations [19].

In light of the International Classification of Diseases, Tenth Revision (ICD-10), congenital anomalies of the ear, eye, face, and neck were the second most common in 44% of the cases [20].

Our study is contributing data on congenital malformations and the associated risks based in Lahore, Pakistan. Data might be available from other countries or other parts of our country, but our objective was to check the prevalence and causes in our locality of Lahore, Pakistan. There were multiple cases being reported every day, and they needed to be investigated. With this data, we can educate the masses against cousin marriages, which are also more prevalent in our society than in the West.

Limitations

This was a retrospective study, and patient files made it difficult to retract all information as records are not digitalized. The study was done on a smaller scale, as more malformations would be prevalent if done with a larger sample size. This was a hospital-based study and may not be projectable to the general population.

## Conclusions

In conclusion, it is evident that both cleft lip and palate have the highest prevalence (110, or 42%) of facial malformations, and parental consanguinity (209, or 80.7%) is one of the leading factors associated with increased risk of facial malformations. This study not only led to the discovery of these causes that have led to the prevalence of cleft lip and palate, but it has also opened many doors for mass education and abundant counseling against cousin marriages and other factors that may lead to these facial anomalies.

There is a need to create public awareness regarding cousin marriages and their link to congenital facial malformations. Protection and prophylaxis against various maternal diseases, such as maternal diabetes and hypertension, may also contribute to fewer craniofacial anomalies. This data and consequent research may be extremely useful, especially if implemented at the community level. Further studies should be done to determine the basic cause of these anomalies.

## Appendices

Appendix A

Questionnaire [21]

Questionnaire for Newborns with Craniofacial Abnormalities

A.    Newborn information 

ID No: _______________________________Age: _____________________________________

Sex: _______________Race: _____________ Mode of delivery  : SVD ____ C-Section _________________ Still birth: _______

Malformation: __________________________________________________________________

Parity: _____________________________________

District: _______________ 

B.    What was the type of marriage? 

  a. Double first cousins 

  b. First cousins 

  c.Second cousins (parents are cousins)

 d. Non-related

C.    Newborn health: Craniofacial abnormality 

a. What is the kind of craniofacial abnormality? ______________________________

b. Is the abnormality unilateral or bilateral? __________________________________

c. Who diagnosed or detected the case? _____________________________________

d. Is there any document or medical report to support the diagnosis? ______________

**D.** ****Investiga**tions**

**Were any of the following investigations carried out during the current pregnancy?** 

                                               i. Calcitonin levels

                                             ii. Blood glucose level 

                                            iii. Hormonal essay 

                                            iv. Alpha-fetoprotein

                                              v. Genetic analysis / karyotyping

                                            vi. B12 levels

                                           vii. Folic acid levels

E.    Maternal health 

a.     During the pregnancy, was the mother taking any medicines? __________

b.     What were the type of medicines? _______________________________

c.     In which period of the pregnancy did she take the medicine?: 

                                               i. First trimester

                                             ii. Second trimester

                                            iii. Third trimester 

                                            iv. Whole duration of the pregnancy

F.    Radiation exposure 

a.     During the pregnancy was she exposed to any type of radiation? Yes ____ No_____

b.     In which period of the pregnancy was she exposed: 

                                               i.First trimester

                                             ii. Second trimester

                                            iii. Third trimester (Whole duration of pregnancy)

G.    Maternal infections

During the current pregnancy did the mother suffer from any infections?

Influenza­­______

Tetanus______

Diphtheria______

Pertussis______

Tuberculosis______

Polio______

Hepatitis______

Human papillomavirus______

Measles______

Mumps______

Meningitis______

Pneumococcal diseases______

Rotavirus______

Rubella______

Yellow fever______

Dengue______

Chicken pox______

Any other______

H.    Smoking history

a. During the current pregnancy did she smoke? Yes _____ No ______

b. In which period of the pregnancy did she smoke? 

                                               i. First trimester

                                             ii. Second trimester

                                            iii. Third trimester

                                            iv. Whole duration of the pregnancy

I.      Alcohol consumption

a.     During the current pregnancy was she drinking alcohol? Yes ___ No ____

b.     If yes, then in which period of the pregnancy did she drink?

                                               i. First trimester

                                             ii. Second trimester

                                            iii. Third trimester

                                            iv. Whole duration of the pregnancy

J.      No. of other family members with congenital anomalies

Relationship type
